# A Protein Isolate from *Moringa oleifera* Leaves Has Hypoglycemic and Antioxidant Effects in Alloxan-Induced Diabetic Mice

**DOI:** 10.3390/molecules22020271

**Published:** 2017-02-11

**Authors:** Paulo C. Paula, Daniele O. B. Sousa, Jose T. A. Oliveira, Ana F. U. Carvalho, Bella G. T. Alves, Mirella L. Pereira, Davi F. Farias, Martonio P. Viana, Flavia A. Santos, Talita C. Morais, Ilka M. Vasconcelos

**Affiliations:** 1 Department of Biochemistry and Molecular Biology, Federal University of Ceara, Fortaleza 60440-900, Brazil; paulolevemir@gmail.com (P.C.P.); jtaolive@ufc.br (J.T.A.O.); bellaagiselly@gmail.com (B.G.T.A.); mirellasally@gmail.com (M.L.P.); davi@dbm.ufpb.br (D.F.F.); vianamp@hotmail.com (M.P.V.); 2 Department of Biology, Federal University of Ceara, Fortaleza 60440-900, Brazil; aurano@ufc.br; 3 Department of Molecular Biology, Federal University of Paraiba, Joao Pessoa 58051-900, Brazil; 4 Department of Physiology and Pharmacology, Federal University of Ceara, Fortaleza 60430-160, Brazil; flavia@ufc.br (F.A.S.); talita.cm@hotmail.com (T.C.M.)

**Keywords:** *Moringa*, plant protein, hypoglycemic activity, antioxidant activity, diabetes therapy

## Abstract

*Moringa oleifera* has been used in traditional medicine to treat diabetes. However, few studies have been conducted to relate its antidiabetic properties to proteins. In this study, a leaf protein isolate was obtained from *M. oleifera* leaves, named *Mo*-LPI, and the hypoglycemic and antioxidant effects on alloxan-induced diabetic mice were assessed. *Mo*-LPI was obtained by aqueous extraction, ammonium sulphate precipitation and dialysis. The electrophoresis profile and proteolytic hydrolysis confirmed its protein nature. *Mo*-LPI showed hemagglutinating activity, cross-reaction with anti-insulin antibodies and precipitation after zinc addition. Single-dose intraperitoneal (i.p.) administration of *Mo*-LPI (500 mg/kg·bw) reduced the blood glucose level (reductions of 34.3%, 60.9% and 66.4% after 1, 3 and 5 h, respectively). The effect of *Mo*-LPI was also evidenced in the repeated dose test with a 56.2% reduction in the blood glucose level on the 7th day after i.p. administration. *Mo*-LPI did not stimulate insulin secretion in diabetic mice. *Mo-*LPI was also effective in reducing the oxidative stress in diabetic mice by a decrease in malondialdehyde level and increase in catalase activity. *Mo*-LPI (2500 mg/kg·bw) did not cause acute toxicity to mice. *Mo*-LPI is a promising alternative or complementary agent to treat diabetes.

## 1. Introduction

Diabetes is a chronic disease that affects millions of people worldwide. The persistent hyperglycemia associated with this disease promotes the appearance of lesions in organs such as kidneys, eyes, nerves and blood vessels. Glycemic control is very important for avoiding or delaying the development of these complications [1]. It is known that several plant compounds exert hypoglycemic effects, representing potential therapeutic agents for the treatment of diabetes [2]. Small molecules with high structural diversity produced during plant secondary metabolism that promote glycemic normalization, stimulate insulin release, reduce resistance to the tissue action of this hormone and elicit insulin-mimetic effects have been isolated, including terpenes, alkaloids and flavonoids [3]. Although a good deal of credit has been given to these secondary metabolites in promoting the hypoglycemic action of orally taken plant extracts, few attention has been paid to observations and data in the literature that relate the antidiabetic properties of plant extracts to proteins [4] and the possibility of their biotechnological use to treat diabetes. In fact, plant proteins have also demonstrated efficient glycemic reduction [5,6]. For example, hypoglycemic proteins were purified from *Canavalia ensiformis* seed coat [7] and *Momordica charantia* seeds [8]. Lectins have also been shown to exert hypoglycemic effects in vivo [9,10,11]. 

*Moringa oleifera* Lamarck (Moringaceae) is a fast-growing perennial species native to northwestern India, which is now cultivated in many areas worldwide. This species has been used in folk medicine to treat diabetes [12,13,14]. Additionally, experimental studies have demonstrated that different parts of the plant possess pharmacological properties related to diabetes, such as hypoglycemic activity [15], anti-inflammatory effects [16], protection against oxidative stress [17] and hypolipidemic activity [18]. The hypoglycemic effect is very often credited to plant secondary metabolites and few efforts have been made to determine whether other molecules possess this activity, especially proteins. Plant secondary metabolites are usually extracted using specific solvents and protocols and during these processes, most proteins lose their structural integrity and activity [19,20]. To the best of our knowledge, there is only one work done on the hypoglycemic activity of proteins from *M. oleifera* [21]. In this study carried out by our research group, a protein fraction obtained from the seed coat of *M. oleifera*, named *Mo*-SC, promoted a substantial reduction in the glucose levels of alloxan-induced diabetic mice after intraperitoneal or oral administration. To expand our knowledge on the biological properties of *M. oleifera* proteins, in the present study a protein isolate was obtained from *M. oleifera* leaves, named *Mo*-LPI (*M. oleifera* leaf protein isolate), and its hypoglycemic and antioxidant effects on alloxan-induced diabetic mice were assessed. To clarify the possible mechanism of hypoglycemic action of *Mo*-LPI, the serum insulin level was evaluated. The results obtained allow to include definitely *M. oleifera* as an additional species within the restricted group of plants that possess proteins with hypoglycemic and antioxidant properties. Accordingly, *M. oleifera* has potential to provide novel protein(s) to compose alternative drug(s) toward treating diabetes.

## 2. Results

### 2.1. Biochemical Properties of Mo-LPI

**Figure 1:**
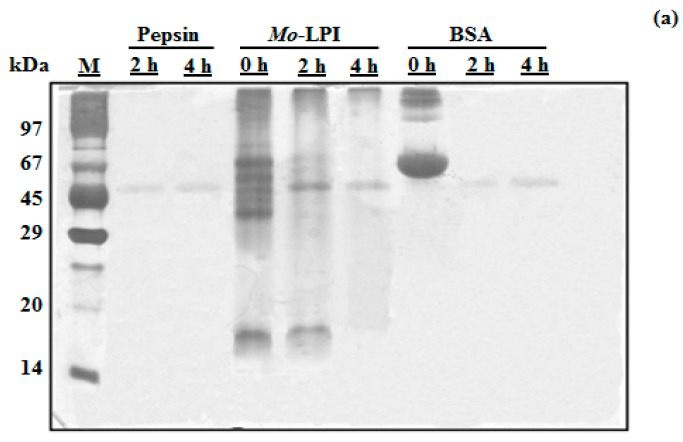

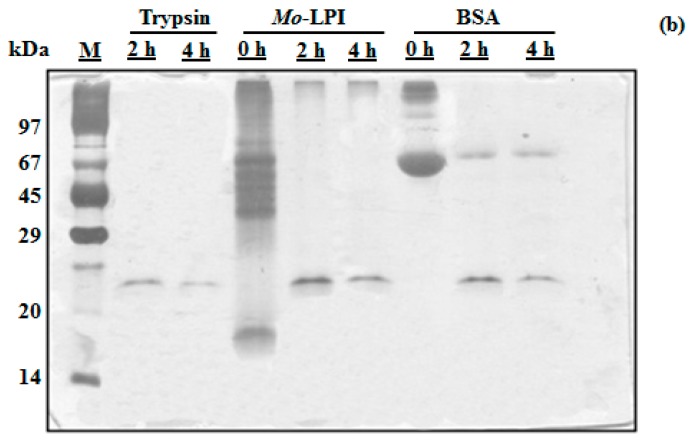
Assessment of the in vitro digestibility of *Mo*-LPI by SDS-PAGE analysis. *Mo*-LPI was incubated with pepsin or trypsin at different times (0, 2 and 4 h). (**a**) Pepsin digestion; (**b**) Trypsin digestion. Bovine serum albumin (BSA) was used as control. Twenty microgram from each sample were loaded in each well. Vertical numbers indicate the molecular mass markers: phosphorylase B (97 kDa); BSA (67 kDa); ovalbumin (45 kDa); carbonic anhydrase (29 kDa); trypsin inhibitor (20.1 kDa) and α-lactalbumin (14.2 kDa). Horizontal numbers refer to the digestion times (h).

*Mo*-LPI corresponds to 4.96 ± 0.33 mg/g leaf dry weight and represents 57.5% of the soluble protein of the leaf crude extract. SDS-PAGE analysis revealed that *Mo*-LPI is composed of several protein bands, most of which have an apparent molecular mass over 29 kDa and a strong single band in the range of 14–20 kDa, as shown in Figure 1 (Lane 4). *Mo*-LPI was susceptible to pepsin digestion and virtually all protein bands disappeared after that (Figure 1a). Similar results were observed after incubation with trypsin; 2-h incubation caused complete digestion of *Mo*-LPI (Figure 1b).

*Mo*-LPI could not agglutinate rabbit erythrocytes, even at 6 mg/mL. However, agglutinating activity was detected against rat (1.33 HU/mg protein) and mouse (2.67 HU/mg protein) erythrocytes. These results did not differ regardless of whether the erythrocytes were trypsin-treated or not.

**Figure 2:**
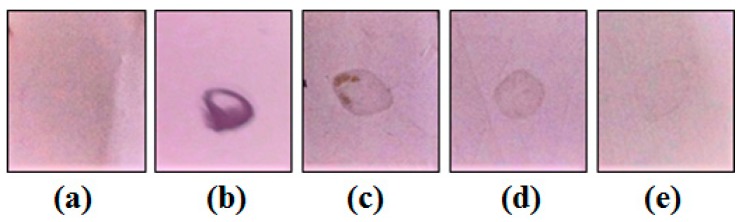
Dot blot assay using human anti-insulin as primary antibodies. (**a**) Tris-buffered saline; (**b**) Human recombinant insulin (2 mg/mL) incubated with human anti-insulin IgG (1:250); (**c**–**e**) *Mo*-LPI (2 mg/mL) incubated with primary antibodies diluted 1:250, 1:500 and 1:1000, respectively.

**Figure 3:**
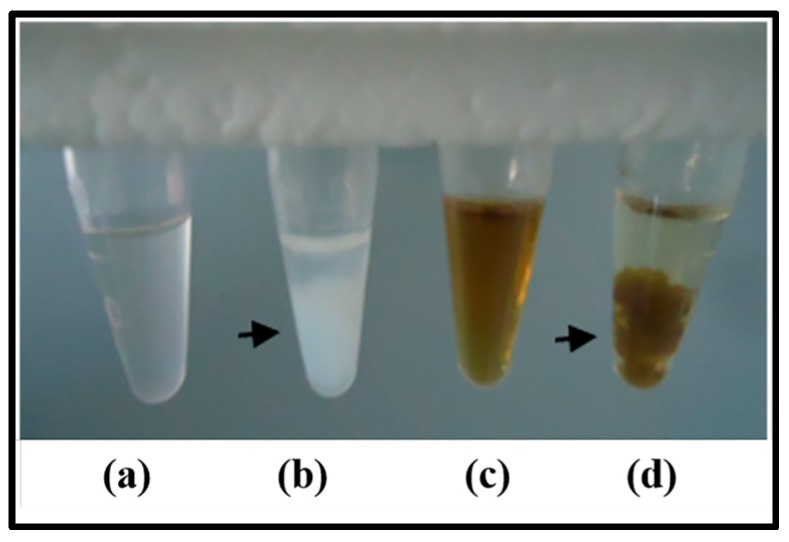
Zn-induced precipitation. (**a**,**c**) zinc-free human recombinant insulin (5 mg/mL) and *Mo*-LPI (5 mg/mL) solutions in 0.05 M Tris-HCl, pH 7.5, respectively; (**b**,**d**) zinc-free human recombinant insulin and *Mo*-LPI solutions after the addition of 1 M zinc chloride, respectively. Arrows indicate the protein precipitate.

*Mo*-LPI was able to cross-react with human anti-insulin antibodies as observed in the dot blot assay. A signal could still be visualized at the lowest primary antibody dilution (Figure 2). Moreover, similar to the effects observed with human recombinant insulin, Zn-induced precipitation of *Mo*-LPI was detected (Figure 3). The proteinaceous nature of this precipitate was confirmed using the Bradford reagent [22].

### 2.2. Effect of Mo-LPI on Fasting Blood Glucose in Alloxan-Induced Diabetic Mice

**Figure 4:**
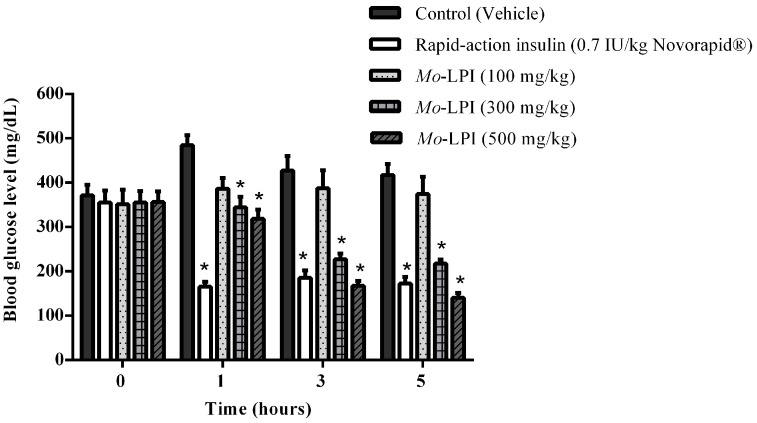
Effect of intraperitoneal administration of *Mo*-LPI on blood glucose level in alloxan-induced diabetic mice. Values are means ± S.E.M. (*n* = 10). Control: vehicle (0.05 M Tris-HCl, pH 7.5, containing 0.15 M NaCl). * Significant (*p* < 0.01) difference when compared with the corresponding value of the control at the same time.

**Figure 5:**
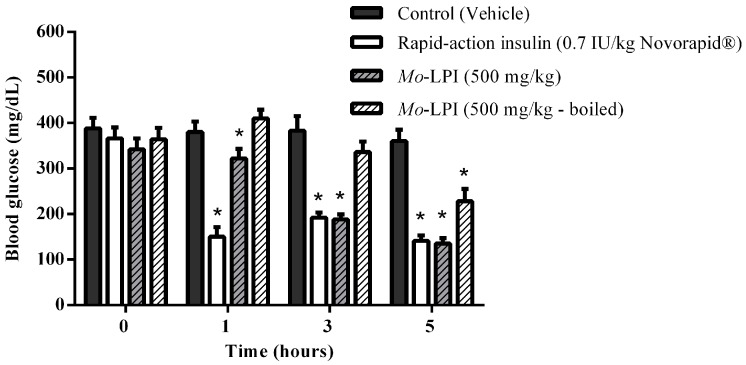
Influence of temperature on the hypoglycemic activity of *Mo*-LPI administered to alloxan-induced diabetic mice by intraperitoneal injection. Values are means ± S.E.M. (*n* = 10). Control: vehicle (0.05 M Tris-HCl, pH 7.5, containing 0.15 M NaCl). *Mo*-LPI unheated and previously boiled to 98 °C for 1 h. * Significant (*p* < 0.01) difference when compared with the corresponding value of the control at the same time.

**Figure 6:**
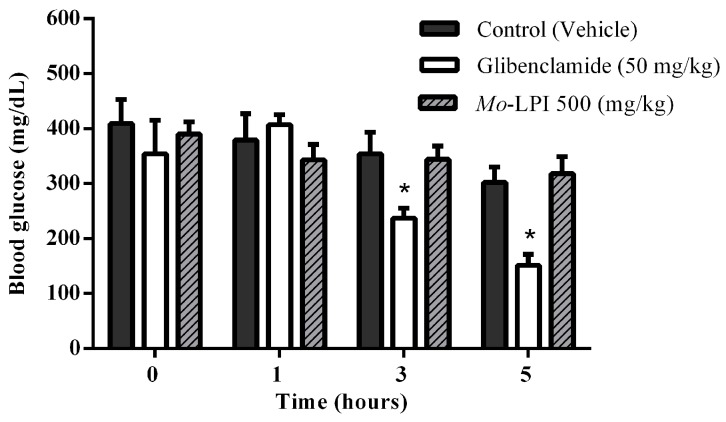
Effect of oral administration of *Mo*-LPI on blood glucose level in alloxan-induced diabetic mice. Values are means ± S.E.M. (*n* = 10). Control: vehicle (0.05 M Tris-HCl, pH 7.5, 0.15 M NaCl). * Significant (*p* < 0.01) difference when compared with the corresponding value of the control at the same time.

The hypoglycemic effect of a single dose of *Mo*-LPI administered to diabetic mice by i.p. injection is shown in Figure 4. *Mo*-LPI potentially and significantly (*p* < 0.01) caused a hypoglycemic effect at both doses (300 and 500 mg/kg·bw). The 500 mg/kg·bw dose presented better antidiabetic activity, with blood glucose reductions of 34.3%, 60.9% and 66.4% after 1, 3 and 5 h, respectively. After heat treatment of *Mo*-LPI (500 mg/kg·bw) at 98 °C for 1 h, the hypoglycemic effect was partially abolished. In this case, the effect was only pronounced at 5 h, with a 36.7% reduction in blood glucose (Figure 5). Conversely, no significant hypoglycemic effect was observed in diabetic mice when *Mo*-LPI was orally administered, even at the highest i.p. dose (Figure 6).

**Figure 7:**
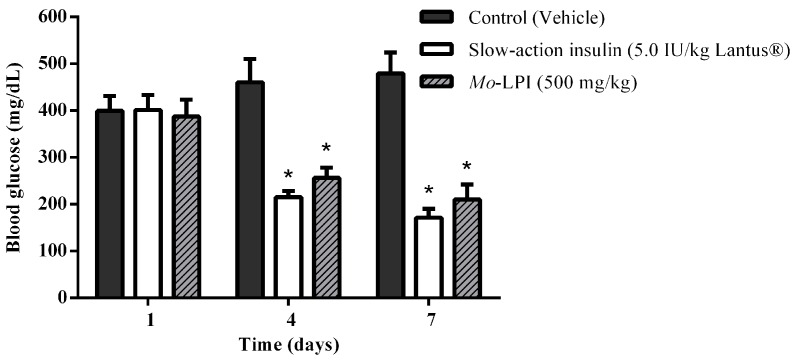
Effect of *Mo*-LPI on blood glucose level after seven consecutive days of intraperitoneal administration to alloxan-induced diabetic mice. Values are means ± S.E.M. (*n* = 10). Control: vehicle (0.05 M Tris-HCl, pH 7.5, 0.15 M NaCl). * Significant (*p* < 0.01) difference when compared with the corresponding value of the diabetic control.

The effect of repeated i.p. administration of *Mo*-LPI on blood glucose levels is presented in Figure 7. When *Mo*-LPI (500 mg/kg·bw) was administered along 7 days to alloxan-induced diabetic mice, a significant (*p* < 0.01) reduction (56.2%) in blood glucose level was observed. *Mo*-LPI exhibited a glucose lowering effect in diabetic mice that was similar to that of insulin.

### 2.3. Effect of Mo-LPI on Serum Insulin Level

**Figure 8:**
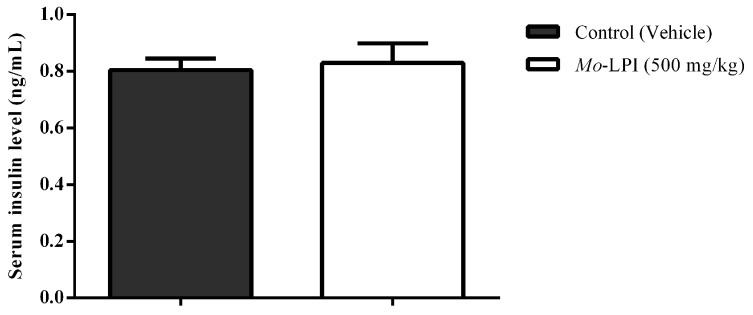
Effect of *Mo*-LPI on serum insulin level in alloxan-induced diabetic mice. Values are means ± S.E.M. (*n* = 10). Five hours after *Mo*-LPI or 0.15 M NaCl i.p. administration in alloxan-diabetic mice, blood samples were collected via cardiac puncture to assay the insulin level. Control: vehicle (0.05 M Tris-HCl, pH 7.5, containing 0.15 M NaCl).

*Mo*-LPI at a dose of 500 mg/kg did not affect the serum insulin level (Figure 8), suggesting that this protein isolate does not stimulate the insulin secretion in diabetic mice.

### 2.4. Effect of Mo-LPI on Lipid Peroxidation and Antioxidant Enzymes in Alloxan-Induced Diabetic Mice

Table 1 shows the effect of *Mo-*LPI on liver lipid peroxidation and antioxidant enzymes in diabetic mice. Significant (*p* < 0.05) increases (71.4% and 82.5%, respectively) in malondialdehyde (MDA) levels were seen in both control and insulin-treated diabetic groups compared to the non-diabetic group. MDA level in diabetic mice treated with *Mo*-LPI was found to be similar to the non-diabetic group. Catalase (CAT) activity increased as a result of *Mo*-LPI administration (56.8%) in comparison to diabetic control. No significant (*p* < 0.05) change of CAT activity was observed in both non-diabetic, diabetic control and insulin-treated diabetic groups. Superoxide dismutase (SOD) activities were similar in diabetic control and *Mo*-LPI-treated diabetic mice. These activities were significantly (*p* < 0.05) lower (73.2% and 68.3%, respectively) when compared with the values recorded in the non-diabetic and insulin-treated diabetic groups. 

### 2.5. Behavioral Effect and Toxicity of Mo-LPI

In the acute toxicity study, no death was reported for 72 h after i.p. administration of *Mo*-LPI at a dose of 2500 mg/kg·bw. In fact, mice treated with *Mo*-LPI did not show any change in their behavioral pattern. There was also no significant difference in body weight or food consumption when compared with the vehicle-treated group (0.05 M Tris-HCl, pH 7.5, containing 0.15 M NaCl). Thus, it was concluded that *Mo*-LPI is safe at 2500 mg/kg·bw, which is five times higher than the dose which was most effective for reducing the blood glucose level.

## 3. Discussion

There are several studies suggesting that plant proteins act as hypoglycemic agents [23,24]. This evidence encouraged us to assess the hypoglycemic effect of *Mo*-LPI, which is considered a pure source of protein, in diabetic mice. The procedure employed to obtain *Mo*-LPI is simple and useful, and basically consists of aqueous extraction, ammonium sulfate precipitation and dialysis, resulting in a good yield of protein: From 1 g of leaf dry weight, approximately 5 mg of *Mo*-LPI can be routinely obtained. The proteinaceous nature of *Mo*-LPI was confirmed by its elevated susceptibility to digestion by pepsin and trypsin. Actually, it has been reported that processing steps such as washing and dialysis minimize the effects of some factors, such trypsin inhibitors, phytates and polyphenols, leading to enhanced digestibility of the plant protein concentrates/isolates [25]. Indeed, *Mo*-LPI is a rich protein source as observed by SDS-PAGE. A lectin is likely to be one of the proteins since *Mo*-LPI agglutinated mouse and rat erythrocytes. This activity of *Mo*-LPI is selective since no agglutination was observed with rabbit erythrocytes. Furthermore, the agglutination activity of *Mo*-LPI could not be further increased by trypsin treatment of erythrocytes. Several plant lectins show similar behavior [26,27]. The importance of detecting lectins in *Mo*-LPI relies on the fact that there are some reports of hypoglycemic activity in vivo of plant hemagglutinins [9,10,11,21].

Another property demonstrated by *Mo*-LPI was its ability to cross-react with an anti-insulin antibody, suggesting the existence of common antigenic epitopes between *Mo*-LPI and insulin. Similar immunoreactivity was observed with proteins isolated from *Canavalia ensiformis* seed coat [7] and *Bauhinia variegata* leaf [28] when in the presence of human anti-insulin antibody. Additionally, *Mo*-LPI showed the same precipitation pattern exhibited by human recombinant insulin after addition of zinc. The zinc-insulin complex is highly insoluble, forming aggregates that precipitate in aqueous solution [29].

To test whether *Mo*-LPI exerts hypoglycemic activity, an alloxan-induced diabetic mouse model was used. Alloxan causes a massive reduction in insulin release because it destroys the β-cells of the islets of Langerhans, inducing hyperglycemia [30]. Thus, the administration of alloxan (150 mg/kg·bw) to the fasted mice markedly increased blood glucose levels. Pharmacological tests confirmed that the i.p. injection of a single dose of *Mo*-LPI into mice reduced significantly (*p* < 0.01) the blood glucose level in a dose-dependent manner. From this study, it could be concluded that *Mo*-LPI at 500 mg/kg·bw caused the maximum reduction in blood glucose, corresponding to 34.3%, 60.9% and 66.4% after 1, 3 and 5 h, respectively; therefore, this dose was selected for further analyses. *Mo*-LPI induced slow and gradual glycemic reductions, whereas insulin caused a rapid reduction (65.9%) in blood glucose within only 1 h of administration. In fact, hypoglycemic events are a fairly common side effect in individuals who use insulin therapy to control diabetes [31,32]. The effect of *Mo*-LPI (500 mg/kg·bw) was also evidenced in the repeated dose test: A 56.2% reduction in the blood glucose level was observed on the 7th day after i.p. administration. Subcutaneous (s.c.) and i.p. administration of a protein (M.Cy) isolated from *Momordica cymbalaria* in rats showed similar percent reductions (66.0% and 69.0%, respectively) in blood glucose [6]. Significant hypoglycemic effects were also observed in rats after i.p. and s.c. administration of a lectin isolated from *Urtica pilulifera* seeds and a protein extract from *M. charantia* fruits, which caused 28.0% and 43.0% reductions in blood glucose, respectively [9,33]. To further characterize *Mo*-LPI, the influence of temperature on its hypoglycemic effect was evaluated. After heat treatment of *Mo*-LPI (500 mg/kg·bw) at 98 °C for 1 h, its hypoglycemic effect was only partially abolished: A 36.7% reduction in blood glucose was observed 5 h after i.p. administration. This result reveals that *Mo*-LPI exhibits resistance to high temperature, suggesting an increased structural stability that may contribute to the retention of its hypoglycemic effect. According to Gifoni et al. [34] and Katre et al. [35], proteins present in *M. oleifera* are often stable at high temperatures and this property could be related to the presence of cysteine residues in their structures.

In contrast, oral administration of *Mo*-LPI (500 mg/kg·bw) did not induce any hypoglycemic effects. It is reasonable to assume that the absence of this effect is caused by proteolysis of *Mo*-LPI along the digestive tract based on its susceptibility to pepsin and trypsin digestion (demonstrated under in vitro conditions). Similarly, the M.Cy protein of *M. cymbalaria* produced a hypoglycemic effect after i.p. administration but not when given orally [6]. In addition, treatment with glibenclamide, an insulin secretagogue, at a dose of 50 mg/kg·bw resulted in 33% and 50% reductions in blood glucose after 3 h and 5 h, respectively, suggesting the presence of functional pancreatic β cells. 

Some plant compounds promote hypoglycemic effect by stimulating the secretion of insulin by the pancreatic β cells [36,37]. This is the mechanism of action of sulfonylureas, a class of antidiabetic drugs widely used in the treatment of type 2 diabetes [38]. In order to evaluate the possible mechanism of action of *Mo*-LPI, its effect on insulin secretion was investigated. *Mo*-LPI showed the hypoglycemic effect without increasing serum insulin in alloxan-induced diabetic mice. Consequently, the mechanism of action of *Mo*-LPI in diabetic mice does not involve stimulation of insulin secretion. Further trials will be conducted in future studies to gather more data about the mechanism of action of *Mo*-LPI.

Increased generation of reactive oxygen species (ROS) is an important aspect in the pathophysiology of diabetes. ROS can damage cellular components, such as proteins, DNA and lipids, resulting in the development of diabetic complications and worsening glycemic control [39]. Products of lipid peroxidation, such as MDA, is elevated with ROS increase being frequently used as markers of oxidative stress. SOD e CAT are important antioxidant enzymes that prevent this process by ROS elimination [40]. Our present study was also undertaken to assess the oxidative status of liver after treatment with *Mo*-LPI by measuring the level of MDA and the activities of SOD and CAT. MDA level in alloxan-induced diabetic mice was found to be higher than that in non-diabetic mice. This could be due to the establishment of chronic hyperglycemia and effect of alloxan [41]. Treatment of diabetic mice with *Mo*-LPI (500 mg/kg·bw; i.p.) in a repeated dose protocol had the ability to prevent lipid peroxidation since the MDA level was close to that observed in the non-diabetic group. In contrast, insulin treatment was not able to reduce MDA levels. It is possible that a transient postprandial hyperglycemia has been more prolonged in the insulin-treated mice than in those treated with *Mo*-LPI. Persistent hyperglycemia can induce ROS generation even after normalizing the blood sugar levels, a phenomenon known as hyperglycemic memory [42]. Regarding antioxidant enzymes, no change of SOD activity between *Mo*-LPI-treated and diabetic control mice was detected. SOD activity in insulin-treated diabetic mice was higher than in those groups. In fact, insulin stimulates SOD activity [43]. As for the CAT, there was no difference between the insulin-treated diabetic and diabetic control groups, whereas *Mo*-LPI-treated animals showed the highest activity. This result may explain the reduction in MDA level promoted by *Mo*-LPI. Hypoglycemic extracts of *Terminalia paniculata*, *Tectona grandis* and *Potentilla discolor* have been shown to restore the antioxidant enzyme activities and normalize or minimize lipid peroxidation in chemical-induced diabetic animals [44,45,46].

To offer more effective and less toxic treatment, any candidate substance to be used as a therapeutic agent must be tested on its toxicity. Thus, a mice acute toxicity assay with *Mo*-LPI was performed. This protein isolate showed no toxicity at a dose of 2500 mg/kg·bw, as no death was reported by 72 h. Additionally, no gross pathological changes were observed. Consequently, the LD_50_ of *Mo*-LPI in mice by i.p. administration is >2500 mg/kg·bw. In contrast, i.p. administration of an aqueous extract of *M. oleifera* leaves has been shown to cause 80% mortality at a dose of 2000 mg/kg·bw [47]. Because *Mo*-LPI is obtained from an aqueous extract of leaves, it is possible that the toxic components were lost during its preparation process.

## 4. Materials and Methods

### 4.1. Plant Material

Fresh *M. oleifera* leaves were harvested from trees naturally growing at Pici Campus from Federal University of Ceara (UFC, Fortaleza, Brazil), throughout the years of 2014 and 2015. Voucher specimens (EAC 54112) were deposited at the Herbário Prisco Bezerra, UFC. *M. oleifera* is an introduced species that is not native from Brazil and thus specific permissions from local authorities to obtain its leaves to be used in the present work were not required. Once harvested, leaves were used to obtain the protein isolate. 

### 4.2. Animals

Conventional male mice, three weeks old, of Biocen-UFC outbred stock originally from Unib:SW (Swiss) (Unicamp, 1965, São Paulo, Brazil) mouse stock were provided by the Central Animal Facility of UFC (Fortaleza, Brazil). The animals were housed at the Department of Biology, at the same University, with temperature (23.0 ± 2.0 °C), photoperiod (12 h of light/12 h of dark) and humidity (45%–55%) monitored. The mice were kept in adequate numbers in polypropylene cages with pine shavings as substrate and water and feed (Biobase, Bio-Tec, São Paulo, Brazil) *ad libitum* until they reach the approximate weight of 30–50 g. 

All experimental procedures were performed in accordance with the current guidelines for the care of laboratory animals (including the use of the 3Rs procedures) and were reviewed and approved by the Animal Ethics Committee (CEPA) of UFC, Brazil (protocol number: 55/2012). This Committee was aware that the possibility of death and suffering of the animals was minimum considering the familiarization with the used protocol and the mice strain.

### 4.3. Obtention of Mo-LPI

*M. oleifera* leaves were washed with distilled water, surface-dried with filter paper and pulverized in liquid nitrogen. The fine powder was extracted in 0.05 M Tris-HCl buffer, pH 8.0, containing 0.15 M NaCl, 2% (*w*/*v*) polyvinylpolypyrrolidone (PVPP), 0.001 M phenylmethysulfonyl fluoride (PMSF) and 0.01 M ethylenediaminetetraacetic acid (EDTA), (1:5, *w*/*v*), under agitation at 4 °C for 30 min. The suspension was filtered through a layer of cheesecloth and the filtrate centrifuged at 15,000× *g* for 30 min at 4 °C. The crude extrac t was precipitated at 90% saturation with ammonium sulfate and allowed to stand overnight. The precipitated proteins were separated by centrifugation as before, dissolved in and dialyzed against distilled water (MW cut-off of 2 kDa) and subsequently lyophilized. This protein isolate (*Mo*-LPI) was used for further analyses.

### 4.4. Protein Determination

Soluble proteins were quantified by the method described by Bradford [22] using bovine serum albumin (BSA) as standard.

### 4.5. In Vitro Digestibility

The in vitro digestibility of *Mo*-LPI was assessed using two proteases commonly found in the gastrointestinal tract of mammals [48], pepsin (EC 3.4.23.1; 2500–3500 U/mg protein, from porcine gastric mucosa) and trypsin (EC 3.4.21.4; 10,000 BAEE U/mg protein, from bovine pancreas). *Mo*-LPI (2 mg) was suspended in 200 µL of 0.1 M HCl, pH 1.8 for pepsin or 0.1 M Tris-HCl, pH 8.1 for trypsin digestion and incubated for 10 min at 37 °C followed by addition of enzyme (1 mg/mL) at a 1:10 enzyme:substrate ratio. The mixtures containing *Mo*-LPI and pepsin or trypsin were incubated in a shaker water bath at 37 °C for 4 h. Aliquots (25 µL) of both enzyme digestions were removed at 0, 2 and 4 h intervals and 25 µL of a 4× concentrated electrophoresis sample buffer (0.125 M Tris-HCl buffer, pH 6.8, containing 0.1% (*w*/*v*) SDS) was added. The digests were immediately heated in boiling water (98 °C) for 5 min and analyzed by SDS-PAGE using a vertical system containing a 12.5% (*w*/*v*) polyacrylamide gel [49]. Electrophoresis was carried out at a constant current of 20 mA. BSA was used as a control to evaluate the enzymatic activity of pepsin and trypsin used. Protein bands were visualized with silver nitrate [50].

### 4.6. Hemagglutinating Activity

Two-fold serial dilutions of *Mo*-LPI (6 mg/mL) in 0.15 M NaCl were incubated with 100 μL of a 2% erythrocyte suspension and cell agglutination was scored after 30 min at 37 °C and an additional 30 min at room temperature (22 ± 3 °C) [51]. Hemagglutinating activity was assayed using rabbit, mouse and rat erythrocytes treated or not treated with trypsin [52]. The minimal protein concentration after serial dilution still promoting visible agglutination with naked eyes was taken to calculate the hemagglutinating activity.

### 4.7. Dot Blot

The presence of proteins in *Mo*-LPI that were able to cross-react with human anti-insulin antibodies was investigated by dot blot analysis [53]. *Mo*-LPI aliquots (20 µL) were applied to a polyvinylidene difluoride (PVDF) membrane. After washing with Tris-buffered saline (0.5 M Tris base, 0.15 M NaCl, pH 8.4) containing 0.05% Tween 20, nonspecific interactions were blocked by incubating the membrane in the same buffer plus 0.05% casein for 1 h at room temperature. The membrane was then washed again and incubated for an additional 3 h at room temperature with serial dilutions (1:250, 1:500 and 1:1000) of human anti-insulin IgG (Sigma-Aldrich, St Louis, MO, USA). Subsequently, a diluted solution (1:2000) of goat anti-rabbit IgG coupled to alkaline phosphatase (Sigma-Aldrich) was added. After 2 h at room temperature, the excess solution was removed by washing and the color reaction developed using 5-bromo-4-chloro-3-indolyl phosphate/nitro blue tetrazolium (BCIP/NBT) as substrate. Human recombinant insulin (Sigma-Aldrich) was used as a positive control and the primary antibody was diluted 1:250.

### 4.8. Evaluation of Potential for Precipitation with Zinc

Zinc-induced precipitation of *Mo*-LPI was evaluated based on previously published data using insulin [54]. *Mo*-LPI (5 mg/mL in 0.05 M Tris-HCl buffer, pH 7.5) was mixed with 1 M zinc chloride at a ratio of 5:2 (*v*/*v*) and incubated for 12 h at room temperature. The same procedure was performed with zinc-free human recombinant insulin (5 mg/mL).

### 4.9. Induction of Experimental Diabetes

Hyperglycemia was induced in mice by a single i.p. injection of 150 mg/kg·bw alloxan monohydrate (Sigma-Aldrich) dissolved in 0.15 M NaCl after 16 h of fasting [55]. Blood samples were collected from the tail vein and the glucose level was determined using a clinical glucometer (AccuCheck Active^®^, Roche, Indianapolis, IN, USA). The blood glucose level was checked before and 72 h after alloxan injection to confirm the development of diabetes. Animals with blood glucose levels ≥300 mg/dL were selected for the study.

### 4.10. Single Dose Test in Alloxan Induced Diabetic Mice

Three sets of experiments were carried out to test the acute hypoglycemic effect of *Mo*-LPI. In all studies, diabetic mice were previously fasted for 4 h and randomly divided into groups of ten animals each. Blood samples were obtained from the tail vein and glucose levels were measured just before treatment (time 0 h) and 1, 3 and 5 h later in all experiments.

In the first experiment, the acute effect of different doses of *Mo*-LPI administered by i.p. injection was examined. The animals were assigned into five groups. Group I (diabetic control or DC): diabetic mice + vehicle (0.05 M Tris-HCl, pH 7.5, containing 0.15 M NaCl); Group II (D + insulin or positive control): diabetic mice + rapid-acting human recombinant insulin (0.7 IU/kg·bw, Novorapid^®^) and Groups III, IV and V (D + *Mo*-LPI): diabetic mice + *Mo*-LPI at doses of 100, 300 and 500 mg/kg·bw, respectively.

In the second test, the influence of temperature on the hypoglycemic activity of *Mo*-LPI was evaluated using the same administration route (i.p.). In this case, four groups were used. Groups I and II were treated as described previously; Group III (D + *Mo*-LPI): diabetic mice + *Mo*-LPI (500 mg/kg·bw) and Group IV (D + *Mo*-LPI boiled): diabetic mice + *Mo*-LPI (500 mg/kg·bw) previously boiled at 98 °C for 1 h. 

Lastly, the third trial was conducted to examine whether *Mo*-LPI displays an oral hypoglycemic effect. Thus, three groups were used. Group I was treated as described previously; Group II (D + glibenclamide or positive control): diabetic mice + glibenclamide (50 mg/kg·bw) and Group III (D + *Mo*-LPI): diabetic mice + *Mo*-LPI (500 mg/kg·bw).

After the last blood glucose measurement in the three sets of experiments, the animals were euthanized by cervical dislocation.

### 4.11. Repeated Dose Test in Alloxan Induced Diabetic Mice

To evaluate the effects of repeated doses of *Mo*-LPI, diabetic animals were treated with the most effective dose identified by the single dose test (500 mg/kg·bw) and subjected to the procedure described by Djomeni et al. [56], with some modifications. Briefly, the freshly prepared solutions were intraperitoneally administered once daily for 7 consecutive days after 4 h of fasting. The diabetic mice were randomly assigned into three groups of ten animals each. Group I (diabetic control or DC): diabetic mice + vehicle (0.05 M Tris-HCl, pH 7.5, containing 0.15 M NaCl); Group II (D + insulin or positive control): diabetic mice + very slow-acting human recombinant insulin (5.0 IU/kg·bw, Lantus®) and Group III (D + *Mo*-LPI): diabetic mice + *Mo*-LPI (500 mg/kg·bw). Following 4 h of fasting, the blood glucose levels were assessed just before and at the end of 3- and 7-day treatment periods. At the end of the experiment, the animals were slightly sedated with halothane and euthanized by cervical dislocation.

### 4.12. Serum Insulin Measurement

Blood glucose was measured 5 h after the *Mo*-LPI administration in a single dose (500 mg/kg·bw, i.p.). The animals were sacrificed by cervical dislocation and blood samples immediately collected via cardiac puncture using a 21 G1 needle attached to a 5 mL syringe. The samples were collected in tubes with no anticoagulant, allowed to stand at room temperature for 30 min and centrifuged at 3000× *g* for 15 min at 4 °C. Serum insulin levels were measured by ELISA, using the rat/mouse insulin ELISA kit (Merk Millipore, Darmstadt, Germany).

### 4.13. Antioxidant Effect Evaluation

At the end of the repeated dose test (Section 4.11), the animals were carefully dissected and the liver used for the assessment of antioxidant activity of *Mo-*LPI. For this, liver samples were homogenized in ice-cold 0.05 M potassium phosphate buffer, pH 7.8, to get a 10% (*w*/*v*) homogenate.

Lipid peroxidation was determined by measuring malondialdehyde (MDA) concentration, following the method proposed by Agar et al. [57]. The liver homogenate obtained was incubated at 37 °C for 1 h and 400 µL of 35% (*v*/*v*) perchloric acid added. After centrifugation at 8300× *g* for 10 min, the supernatant was collected and 200 µL of 1.2% (*v*/*v*) thiobarbituric acid added. The resulting mixture was further incubated at 95 °C for 30 min and aliquots were removed to measure absorbance at 532 nm in a microplate reader (Expert Plus Analytical, (Biochrom, Cambridge, UK)). A standard curve was built with known concentrations of 1,1,3,3-tetramethoxypropane and results expressed as micromoles of MDA produced per gram of tissue (µmol/g tissue).

Catalase (CAT) activity was measured according to the method described by Aebi [58]. The reaction mixture consisted of 20 µL of 5% (*w*/*v*) liver homogenate and 2 mL of 0.05 M potassium phosphate buffer, pH 7.8, containing 0.01 M H_2_O_2_. Immediately thereafter, the absorbance at 230 nm was recorded. Catalase activity was expressed as units/µg protein, where one unit is defined as the amount of enzyme required to decompose 1 nmol H_2_O_2_ per min at 25 °C.

Superoxide dismutase activity (SOD) was determined as described by Beauchamp and Fridovich [59], with modifications. The liver homogenate obtained was centrifuged at 12,000× *g* for 20 min at 4 °C and the supernatant collected. In a darkroom, 10 µL of this supernatant, 1 mL reaction medium (0.05 M potassium phosphate buffer, pH 7.8, 0.1 nM ethylenediamine tetraacetic acid and 0.013 mM L-methionine), 150 µL of 0.75 µM NBT and 300 µL of 2 µM riboflavin were mixed. The mixture was exposed to fluorescent light (15 W, 15 min) followed by absorbance measurements at 560 nm. The results were expressed as units/µg protein, where one unit of SOD is defined as the amount that inhibits the NBT photoreduction by 50%.

### 4.14. Acute Toxicity Assessment

Ten mice (male and female) were treated (i.p.) with *Mo*-LPI at a dose of 2500 mg/kg·bw. The animals were continuously observed up to 4 h and subsequently at 24 and 72 h after administration and signs of toxicity and mortality were recorded [60].

### 4.15. Statistical Analysis

The results are expressed as means ± S.E.M. The statistical analysis was performed by one-way analysis of variance (ANOVA) followed by Tukey’s multiple comparison test. However, in the assay for measurement of serum insulin, the results were analyzed using Student’s *t*-test (two unpaired samples). The results were considered to be significantly different at *p* < 0.01 or *p* < 0.05.

## 5. Conclusions

The findings of this study allow us to conclude that *Mo-*LPI has hypoglycemic effect in alloxan-induced diabetic mice and that its mode of action does not involve a secretagogue stimulation of insulin. *Mo-*LPI was also effective in reducing the oxidative stress in mice associated with diabetes. Moreover, *Mo*-LPI did not cause acute toxicity to mice, even at high dose, suggesting its potential for safe biotechnological applications. Taken together, these data support the use of proteins from *M. oleifera* leaves as an alternative or complementary agent to treat diabetes.

## Figures and Tables

**Table 1:** Malondialdehyde levels and antioxidant enzyme activities in hepatic tissues of non-diabetic and alloxan-induced diabetic mice.

Group	MDA (μmol/g Tissue)	CAT (U/µg Protein)	SOD (U/µg Protein)
Diabetic control	57.43 ± 3.48 ^a^	1.92 ± 0.18 ^a^	0.42 ± 0.02 ^a^
Non-diabetic	33.50 ± 4.23 ^b^	1.88 ± 0.23 ^a^	1.42 ± 0.06 ^b^
d + Insulin (0.7 IU/kg)	61.13 ± 3.58 ^a^	2.01 ± 0.16 ^a^	1.20 ± 0.04 ^c^
d + *Mo*-LPI (500 mg/kg)	27.78 ± 2.15 ^b^	3.01 ± 0.35 ^b^	0.38 ± 0.02 ^a^

Values are means ± S.E.M. (*n* = 10). Diabetic control: vehicle (0.05 M Tris-HCl, pH 7.5, containing 0.15 M NaCl); Non-diabetic: vehicle (0.05 M Tris-HCl, pH 7.5, containing 0.15 M NaCl); d + insulin: diabetic mice + rapid-acting human recombinant insulin (0.7 IU/kg·bw, Novorapid^®^); d + *Mo*-LPI: diabetic mice + *Mo*-LPI (500 mg/kg·bw). Different letters in the same column indicate significant differences (*p* < 0.05) when compared with the corresponding value of the diabetic control.
